# Psychological Help-Seeking Status for Living with Mental Health Conditions in Türkiye: Using a Multivariate Probit Model

**DOI:** 10.3390/healthcare11212837

**Published:** 2023-10-27

**Authors:** Esra Bayrakçeken, Ömer Alkan, Hayri Abar

**Affiliations:** 1 Department of Medical Services and Techniques, Vocational School of Health Services, Ataturk University, Erzurum 25030, Türkiye; esra.bayrakceken@atauni.edu.tr; 2 Department of Econometrics, Faculty of Economics and Administrative Sciences, 2nd Floor, Number 222, Ataturk University, Erzurum 25030, Türkiye; 3 Master Araştırma Eğitim ve Danışmanlık Hizmetleri Ltd., Şti., Ata Teknokent, Erzurum 25240, Türkiye; 4 Department of Economics, Faculty of Business and Economics, Gaziantep University, 1st Floor, Number 132, Gaziantep 27310, Türkiye; hayriabar@gmail.com

**Keywords:** psychological stresses, factor, psychological mental health, regression, Türkiye

## Abstract

Background/aim: Mental disorders constitute a significant public health concern, contributing significantly to the overall disease burden. Individuals experiencing mental distress often delay or avoid seeking psychological help or do not seek help due to varying factors. This study examines the factors affecting individuals’ preferences, particularly young people, in seeking psychological help in Türkiye. Methods: This study utilized microdata from the Türkiye Health Survey conducted by TURKSTAT in 2016 and 2019. The factors influencing decisions to consult with a psychologist, psychotherapist, or psychiatrist were determined by multivariate probit regression analysis. The Türkiye Health Survey employed a stratified two-stage cluster sampling method, surveying 17,242 individuals over the age of 15 in 9470 households in 2016 and 17,084 individuals over the age of 15 in 9470 households in 2019. Results: The analysis findings indicated that, in the entire sample, women are more likely to seek psychological help, while younger women are less likely to do so. In the entire sample, as the education level increased, the probability of individuals receiving psychological help increased, while it decreased for young people. It was determined that people who use tobacco and alcohol are more likely to receive psychological help than others. It was also determined that individuals who perceive their health status as good, especially those who are both young and perceive their health status as good, are less likely to seek psychological help. Conclusions: The significance and impact of the variables on the probability of individuals seeking psychological help vary for both the entire sample and young people. There may be various restrictions in getting psychological help, and people may resort to informal methods instead of professionals to cope with their existing problems. In developing preventive strategies to safeguard mental health, factors related to young people’s and individuals’ psychological help-seeking status should be prioritized, and more attention should be paid to them.

## 1. Introduction

Mental disorders, a significant public health issue, account for 10% of all diseases that cause death or disability globally and 30% of all non-fatal diseases [1]. The most common mental health problems in society are anxiety and depressive disorders. More than 300 million people worldwide, or 4.4% of the population, are believed to experience depression [2]. The prevalence of depression in Türkiye was 9.0% [3], somatization disorder prevalence was 5.0%, and panic disorder prevalence was 2.0% [4]. From 2002 to 2019, years of life spent with disability due to depressive and anxiety disorders increased in Türkiye by 22% [3]. Most mental health problems occur in late childhood and early adolescence. Mental disorders contribute to most of the disease burden in individuals aged ten to twenty-four and are also the most important cause of disability for this age group [5,6]. Almost two-thirds of the years of life lost to disability in young people between the ages of fifteen and twenty-four are due to mental health disorders, substance use, murder, and suicide, and each of these variables has a strong relationship with mental health. Unipolar and bipolar depressive disorders, schizophrenia, violence, self-mutilation, panic disorders, and alcohol use constitute 7 of the top 10 causes of disability-adjusted life years (DALYs) in young people aged 15–24. Every year, 800,000 young people between the ages of 15–29 and 53,000 young people between the ages of 15–19 die due to suicide [7].

People may seek help to cope with the mental problems that they experience. Help-seeking, defined as seeking a solution to meet a need, has various definitions [8]. It is characterized as individuals going through a distressing process, reaching out to others for advice, information, treatment, or general support [9]. Generally, help-seeking is the process by which a person seeks resources to address self-identified needs, such as experiences of violence, health problems, and financial difficulties [10]. Help-seeking behaviour involves communicating with others to obtain help through advice, information, treatment, and general support for problem resolution. There are formal and informal types of help-seeking. Mental health professionals provide formal help, while family, friends, and others offer informal help [11].

Going to a doctor for psychological support or treatment may be a choice when seeking help for mental health problems. The prevention and treatment of mental health issues require professional support [12]. Psychological support methods can be classified according to the theoretical model, treatment object, treatment method, and duration. According to the theoretical model, psychological support can be categorized as analytical psychological therapy, cognitive psychotherapy, supportive psychotherapy, behavioural psychotherapy, and interpersonal psychotherapy [13]. In Türkiye, clinical psychologists, psychologists, social workers, and nurses contribute to public health protection and the prevention of mental health issues [14,15]. However, psychiatrists and clinical psychologists dominate the mental health field in Türkiye. State-sponsored mental health services are primarily provided in public hospitals and mental health institutes that offer psychiatry and social assistance [16]. The Ministry of Health of the Republic of Türkiye also operates community mental health centres that provide psychosocial support services for patients with chronic mental disorders such as schizophrenia, mood disorders, and similar conditions. These centres monitor and treat patients as needed and provide social rehabilitation [17]. In Türkiye, people who have completed their medical education and specialized in psychiatry are referred to as psychiatrists. Psychologists are those who have graduated from the psychology department of 4-year faculties. Those who complete their master’s and doctoral education in clinical psychology also become clinical psychologists. Psychiatrists or clinical psychologists perform psychotherapy. Psychologists, in addition to providing psychological support and services, conduct interviews with patients and prepare reports using the tests, techniques, and methods they have learned through training deemed appropriate by the ministry, under the supervision of a clinical psychologist [18,19].

Although preventive and therapeutic services for mental health problems, a leading cause of mortality and morbidity worldwide, are increasing, disease incidence has not decreased [20]. This is partly because mental health is threatened by various social, economic, and environmental issues that are frequently overlooked or ignored [1]. According to the Turkish Mental Health Profile research, psychiatrists are the most common first point of reference [21]. According to the Social Ecological Model, many factors influence help-seeking behaviours, including individual, interpersonal and social factors [22]. 

## 2. Literature Review

People are exposed to social, economic, and ecological risks throughout their lives, negatively affecting their mental health. Studies have reported that the lack of social support and financial difficulties [23,24] can cause psychological issues. While long-term unemployment causes anxiety and stress even in young people [25], it also affects young people’s behaviour in seeking psychological help [26]. On the other hand, psychological problems such as depression negatively affect people’s working capacity and economic stability [27]. Additionally, life-period risk factors such as substance abuse [28], fear and distress [29], pandemics [30], migrations and old age [31], pregnancy [32], poverty [33], and physical diseases leading to disability [34] can also impair the mental health of individuals. 

Mental health problems limit people, can lead to mental disabilities [35], and elevate the risk of suicide [36,37]. Suicidal thoughts and attempts are reported to be high among young people in Turkey [38]. Delaying or avoiding psychological help may result in negative consequences, such as substance abuse, risky sexual behaviours, and a declining quality of life [39]. Mental health problems seen in young people can manifest themselves in different ways later in life. These health problems may cause an increase in the burden of disease, an increase in disability-adjusted life years (DALYs), the loss of productivity, and an increase in financial expenses for the individual and family; cigarette, alcohol, and addictive substance use; mood, personality, behaviour, and sleep problems; and suicide attempts [7]. 

Mental health and psychological well-being are essential for individuals to form and maintain relationships, pursue interests in school, work, or leisure, and lead fulfilling lives. This includes making everyday decisions on employment, housing, and education [40]. To protect people from the effects of mental health problems, they must receive treatment as soon as possible. Despite negative attitudes towards seeking professional psychiatric help, inadequate or delayed help-seeking behaviour can impede treatment effectiveness [12]. Individuals may not be protected from the consequences of mental health disease if treatment is delayed. Factors such as culture [41], self-stigmatization [12,42], past personal experiences, and belief in treatment [43] can hinder individuals from seeking psychological help. Additionally, it has been reported that gender, age, social support (employment status and family function), and depression literacy are also significantly associated with attitudes toward seeking professional psychological help [12]. In a study conducted on Arab youth, it was determined that young people had negative attitudes towards seeking psychological help [44]. It was also stated that young women were more active in seeking psychological help [45]. Even if individuals exhibit symptoms, they may be hesitant to seek psychological counselling due to a lack of confidence in the benefits of counselling [46]. In addition, young individuals’ low mental health literacy can pose an obstacle to seeking psychological help. Even if young individuals have serious mental health problems, they turn to friends, parents, and the Internet instead of seeking professional services [47]. The reluctance of young individuals to seek help increases their likelihood of living with psychological difficulties [48].

When examining psychological help-seeking behaviours in Türkiye, research has found that women tend to have more positive attitudes towards seeking psychological help than men [11]. Men have been found to harbour stronger negative feelings of self-stigma related to seeking psychological help [49]. Additionally, individuals’ perceptions of social support can affect their decision to seek psychological help. Studies suggest that the support of friends, in particular, can impact an individual’s help-seeking behaviours [11]. A study conducted in Türkiye found that exposure to noise, violence, and crime in the neighbourhood impairs women’s mental health, while employment issues adversely affect men’s mental health. Other factors that have been found to impact mental health include income, overall health, lack of trustworthy confidantes, impolite behaviour, and discrimination in the workplace [23]. A study conducted with a group of young people in Türkiye found that 15 of 100 participants had considered suicide at least once and that the deterioration of both physical and mental health exacerbated the intensity of mental symptoms [50]. 

In Türkiye, individuals’ choices to see a psychologist, psychotherapist, and psychiatrist are affected by many factors. Knowing these factors is vital in planning individual interventions and determining support sources. Studies on this subject in Türkiye are limited. Therefore, this study aimed at finding an answer to the question “What are the factors affecting the preferences of individuals living in Turkey to seek help from a psychologist, psychotherapist or psychiatrist?”.

Studies in this area in Türkiye are limited; thus, this study aimed to answer the question “What are the factors that affect individuals living in Türkiye to seek help from a psychologist, psychotherapist or psychiatrist?”. The factors affecting individuals’ seeking psychological help services were modelled for Türkiye using a rich data set.

The following are the research questions in this study that focus on whether individuals and young people living in Türkiye receive help from psychologists, psychotherapists, and psychiatrists: 

Research Question 1: What is the prevalence of receiving help from psychologists, psychotherapists, and psychiatrists for individuals living in Türkiye?

Research Question 2: Are sociodemographic, economic, and other factors related to receiving help from psychologists, psychotherapists, and psychiatrists for individuals, especially young individuals living in Türkiye? 

Research Question 3: Are the individuals’ propensity to receive help from a psychologist, psychotherapist, and psychiatrist interrelated?

## 3. Material and Methods

This section explains the details of the data used in the study, the dependent and independent variables, and the research method.

### 3.1. Data

This study used the microdata set from the 2016 and 2019 Türkiye Health Surveys conducted by TURKSTAT. TURKSTAT has shared the latest data for 2019. This research, which provides an overview of the country’s general health status, is significant as it is the first of its kind and highlights the need for international and national comparisons. The Health Survey provides information on various health indicators, including the health status of infants, children, and adults, individuals’ use of health services, any difficulties they may experience with daily activities, and their smoking and alcohol consumption habits [51,52].

The Türkiye Health Survey aims to get information about health indicators, which constitute a significant part of the development indicators reflecting a country’s level of development. This survey is crucial as it is the first study that provides a comprehensive view of the country and addresses both international and national needs, allowing for meaningful comparisons. It covers all individuals living in Türkiye. The institutional population (soldiers, individuals living in dormitories, prisons, hospitals in the long term, homes for older individuals, etc.) and individuals living in small villages, hamlets, etc. (fewer than 20 addresses) are excluded [51,52].

A stratified two-stage cluster sampling method was used in the Türkiye Health Survey. As the criterion for external stratification, rural-urban separation was used (settlements with a population of 20,000 or less and settlements with a population of 20,001 or above were considered urban). The first stage sampling unit consisted of randomly selected blocks with a selection proportional to the size of the clusters (blocks), each containing an average of 100 addresses. The second stage sampling unit involved systematically randomly selected household addresses from each selected cluster [51,52].

The sample design consisted of two phases. In the first phase, 947 clusters were selected as the “first phase sampling unit”. These 947 houses comprised 711 urban-rural, 118 urban-rural, and 118 rural-rural settlements. In the second phase, as a “second phase sampling unit”, considering intra-cluster homogeneity coefficients, ten address samples were selected from each of the 947 clusters, resulting in a total sample volume of 9470 household addresses. The Türkiye Health Survey in 2016 was applied to 17,242 people over the age of 15, and the Türkiye Health Survey in 2019 was applied to 17,084 people over the age of 15. Data from 34,326 individuals were used in the analysis [51,52].

### 3.2. Dependent Variables

In the Türkiye Health Survey, participants were asked, “Have you seen a psychologist for yourself in the last 12 months?”, “Have you gone to a psychotherapist for yourself in the last 12 months?”, and “Have you seen a psychiatrist for yourself in the last 12 months?”. The answers to these questions consisted of two categories: yes and no. If individuals answered yes to whether they received psychological support, they received the code 1; if they answered no, they received the code 0. The study’s dependent variables were whether or not they went to a psychologist in the last 12 months, whether or not they went to a psychotherapist, and whether or not they went to a psychiatrist.

The frequencies and percentages of the study’s dependent variables are presented in Table 1. According to Table 1, 2.4% of people consulted a psychologist, 0.8% consulted a psychotherapist, and 4.2% consulted a psychiatrist in the last year. 

### 3.3. Independent Variables

There are numerous variables related to health in the Türkiye Health Survey. In this study, only those variables that could affect the occupational groups individuals turn to when seeking psychological help were utilized. Therefore, the independent factors in the study are believed to be influential in the decision to seek psychological help. Independent variables were selected based on the existing literature as much as possible. The independent variables in this research include survey year (2016, 2019), gender (female, male) [11,12], age (15–24, 25 and over) [53], treatment costs covered by SSI, bond, pension fund, or general health insurance (yes, no) [24], education level (illiterate/no degree, primary school, elementary school, high school, university) [54], marital status (married, single), employment status (employed, not employed) [24], general health status (very good/good, moderate, poor/very bad), sickness/health problem lasting/expected to be lasting six months or longer (yes, no) [55], heart attack in the last 12 months (yes, no), inability to afford the prescribed medication due to failure constraints (yes, no), tobacco use (yes, no), alcohol use (yes, no), having someone to trust in case of severe personal problems (none, 1–2 people, three or more people), and receiving help from neighbours in case of need (very easy/easy, moderate, hard/very hard) [56,57]. The same independent variables were used in the youth.

Survey year, gender, treatment costs covered by SSI/bond/pension fund/general health insurance, marital status, employment status, sickness/health problem lasting/expected to last six months or longer, heart attack in the last 12 months, inability to afford the prescribed medication due to failure to pay, tobacco use, and alcohol use were variables measured with a nominal scale. Age, education level, general health status, having someone to trust in case of serious personal problems, and getting help from neighbours in case of need were variables measured on an ordinal scale.

Since a large sample reflecting the entire country was used in the study, many independent variables were used instead of focusing on the critical factors affecting the dependent variables to make a general evaluation. Since the number of observations is large enough, adding too many variables does not pose a methodological problem.

### 3.4. Statistical Analysis

The data analysis was conducted using SPSS (IBM SPSS Statistics 20.0), Stata (Version 14), and R software (Version 3.0.1). SPSS (IBM SPSS Statistics 20.0) was used to calculate descriptive statistics, Stata (Version 14) was used to estimate the model, and R (Version 3.0.1) was used to calculate marginal effects. The study first determined the preferences for psychological help among the participating individuals, along with their frequencies and percentages based on the independent variables. Subsequently, through multivariate probit regression analysis, the study identified the factors that were influential in seeking psychological help. 

The multivariate probit model is preferred when the dependent variables are related [58,59]. In terms of the relationship between dependent variables affected by the same explanatory variables, the multivariate probit model provides more accurate results than binary logit or probit models for each dependent variable separately [60]. One of the most important uses of the multivariate probit model is that it can demonstrate the interaction between the dependent variables.

The general definition for the multivariate probit regression model is as follows:

(1) $$Y_{im}^{*}=x_{im}^{\prime }\beta_{m}+\varepsilon_{im}\;Y_{im}=1\;if\;Y_{im}^{*}>0\;and\;0\;otherwise\;for,\;i=1,2,\ldots N;\;m=1,\;2,3$$

$$E\left(\varepsilon_{im}|x_{i1},\ldots ,x_{im}\right)=0$$

$$Var\left(\varepsilon_{im}|x_{i1},\ldots ,x_{im}\right)=1$$

$$Cov\left({\varepsilon_{ij},\varepsilon }_{im}|x_{i1},\;x_{i2},\ldots ,x_{im}\right)=\rho_{jm}$$

$$\left(\varepsilon_{i1},\;\ldots ,\varepsilon_{im}\sim N_{m}\left[0,\;R\right]\right)$$

where *X* is the covariate matrix with any explanatory variable, *β* is the unknown regression coefficient matrix, *ε* is the error term, *R* is the variance-covariance matrix, and ρ_jm is the correlation matrix [61].

Categorical variables can be included in models using dummy variables. Dummy variables indicate the presence or absence of a particular attribute. They take the value 1 when the attribute of interest is present and 0 when it is not. To investigate the effect of the combination of specific categories of two categorical variables, the interaction of the categorical variables, i.e., the multiplication of the categorical variables, is included in the model as a new independent variable. This allows us to reveal the difference between units that have both attributes of interest and those that do not. In this study, a dummy variable defined as “being youth” was included in the model to highlight the difference between youths and non-youth individuals seeking psychological help services. Additionally, the youth dummy variable was incorporated into the model by multiplying it with other independent variables to determine the effect of the interaction between being a youth and other attributes. 

## 4. Results

### 4.1. Descriptive Statistics

According to Table 2, 55% of the participants in the study were female, 16.4% were between the ages of 15 and 24, and 83.6% were over 25 years old. It was determined that the social security institution covered 88.1% of the participants who participated in the survey. Additionally, 33.7% were illiterate or did not graduate, while 16.4% were high school graduates. The data showed that 68.9% of the research group were married, 62.2% were unemployed, and 59% reported having a very good/good general health condition. Furthermore, 54.8% reported having a disease, 2.5% had a heart attack, and 10.5% faced difficulty with health payments. Additionally, 30% of participants reported using tobacco, and 13.4% reported using alcohol. Moreover, 5.5% of the participants in the study group stated that they never had a trusted relative, while 72.1% of the individuals reported that they could quickly or very quickly request assistance when needed. Finally, 12.5% expressed a need for help when requesting aid.

### 4.2. Model Estimation

The multivariate probit model was used to identify the variables affecting the psychological help-seeking decisions of the individuals included in the study. If individuals and youths had visited any psychologists, psychotherapists, or psychiatrists, they were considered to have sought psychological help. The results of the multivariate probit regression analysis conducted to identify the factors affecting the decision to seek psychological support are provided in Table 3. The independent variables used in the study are not standardized since they are categorical.

According to Table 3, factors such as gender, survey year, education, marital status, employment status, general health and illness status, having a heart attack, difficulties with health payments, tobacco and alcohol use, as well as seeking help from a reliable relative or neighbour all play a role in affecting individuals’ decisions to seek psychological help. Conversely, in the case of youths, gender, survey year, being between the ages of 15–24, education, alcohol use, and having a reliable relative and a neighbour from whom he/she can get help had an effect on seeking psychological help in youths.

### 4.3. Marginal Effects

Each category of independent variables with more than two categories was represented by a dummy variable in the multivariate probit regression model. This allowed us to interpret the changes in the model caused by the changes in each category through marginal effects while holding other variables constant.

For the multivariate probit model, the coefficients indicate the effect of the independent variable on the z-score or multivariate probit index. Marginal effects can be used to make more understandable interpretations. These effects demonstrate how the predicted probability of the binary dependent variable changes in response to alterations in the dependent variables. Since the marginal effects differ according to each observation point in the multivariate probit model, the marginal effects can be calculated as many as the number of observations. To provide a comprehensive interpretation, these marginal effects are averaged, and the average marginal effect is calculated. In this study, the average marginal effects presented in Table 4 are interpreted.

Three models were estimated in the study. An independent variable may not be significant for all dependent variables. When interpreting the model, only significant independent variable coefficients were interpreted.

In Table 4, the marginal effects of demographic and socioeconomic factors affecting individuals’ decisions to seek psychological help are presented. The results show that women are more likely than men to visit a psychologist, psychotherapist, and psychiatrist by 1.58%, 0.34%, and 2.20%, respectively. Individuals who participated in the study in 2019 are 0.76% more likely to visit a psychiatrist. Primary, elementary, high school, and university graduates are 1.39%, 1.46%, 0.08%, and 2.92% more likely to visit a psychologist than those who are illiterate/did not have formal education, respectively. Similarly, those who completed primary, high school, and have a university education are 0.66%, 1.55%, and 1.66% more likely to visit a psychotherapist. Primary, elementary, high school, and university graduates are 2.61%, 3.82%, 4.46%, and 5.11% more likely to visit a psychiatrist, respectively. Compared to singles, married people are 0.55%, 0.30%, and 1.16% less likely to visit a psychologist, psychotherapist, or psychiatrist, respectively. Employed individuals are 1.08% less likely to visit a psychiatrist than those not employed.

Those with good/very good general health are 1.88%, 0.55%, and 3.46% less likely to visit a psychologist, psychotherapist, and psychiatrist, respectively, compared to those with poor/very poor health conditions. Compared to those with poor/very bad health, those with moderate general health status are 0.92%, 0.35%, and 1.71% less likely to visit a psychologist, psychotherapist, and psychiatrist, respectively. Those with heart attacks are 1.46% and 0.84% less likely to visit a psychologist and psychotherapist, respectively. Individuals who use tobacco are 0.87%, 0.36%, and 1.90% more likely to visit a psychologist, psychotherapist, and psychiatrist, respectively. People who drink alcohol are 0.56% and 0.97% more likely to visit a psychologist and psychiatrist. Those who do not have a reliable relative are 1.56% and 0.77% more likely to visit a psychologist and psychotherapist than those with three or more reliable relatives, respectively. Those with 1–2 reliable relatives are 0.48%, 0.43%, and 0.50% more likely to visit a psychologist, psychotherapist, and psychiatrist compared to those with three or more reliable relatives, respectively.

Those between the ages of 15 and 25 are 8.34% and 9.28% more likely to visit a psychologist and psychiatrist, respectively, compared to those aged 25 and older. Young women are less likely than others to visit a psychologist or psychiatrist, by 1.12% and 1.45%, respectively. In 2019, young people were 1.15% less likely to visit a psychiatrist. Youths with a primary school degree are 2.19% and 0.59% less likely to visit a psychologist and psychotherapist, respectively, compared to those who have not completed any school and/or are not young. Youths with a primary school degree are 0.71% and 2.24% less likely to visit a psychotherapist and psychiatrist, respectively. Youths with a high school degree are 0.77% less likely to visit a psychotherapist than those who have not completed any school and/or are not young. Youths with a bachelor’s degree are 1.45% and 0.70% less likely to visit a psychologist and psychotherapist, respectively, compared to those who have not completed any school and/or are not young. Youths who have suffered a heart attack are 0.77% less likely to visit a psychotherapist than others. Young individuals facing challenges in paying for health care are 0.44% less likely to visit a psychotherapist. Youths who consume alcohol are 2.78% more likely to visit a psychiatrist compared to others. Youths who do not have a reliable relative are 0.78% less likely to visit a psychotherapist than those with three or more reliable relatives and/or are not young. Those with 1–2 reliable relatives are 0.76% less likely to visit a psychologist than those with three or more reliable relatives and/or are not young. Youths who easily receive help from their neighbours are 1.06% and 0.41% less likely to visit a psychologist and psychotherapist, respectively, compared to those who receive difficult/very difficult help from their neighbours and/or are not young. Youths who receive help from their neighbours at a moderate level are 1.45% less likely to visit a psychologist compared to those who receive difficult/very difficult help and/or are not young.

One of the most important uses of the multivariate probit model is that it can demonstrate the interaction between the dependent variables. The estimated correlation coefficients between the dependent variables are provided in Table 5. According to these coefficients, a positive relationship exists among the dependent variables, allowing them to be collectively considered simultaneously in a single model.

## 5. Discussion

This study aimed to investigate the factors affecting the decision of individuals in Türkiye to seek psychological help from psychologists, psychotherapists, and psychiatrists. The results indicated that gender, age, survey year, education, marital status, employment status, general health and disease status, history of a heart attack, difficulty paying for health care, tobacco and alcohol use, having a reliable relative, and ease of receiving help from neighbours all affected the behaviour of seeking psychological help in Turkish society. Regarding the behaviours of youths between the ages of 15–24 in Türkiye, seeking psychological help, gender, survey year, education, alcohol use, having a reliable relative, and having a neighbour from whom they can seek help were also significant factors.

The study results indicated that gender is a significant factor in the psychological help-seeking behaviour of individuals living in Türkiye. Although the psychological help-seeking behaviour of women in the entire sample is greater than that of men, young women exhibit lower rates than young men. The help-seeking behaviour of women in the entire sample aligns with findings in the literature [11,12]. Evidence from the literature indicates that young women may be more proactive in seeking psychological help [45]. Additionally, studies conducted during the pandemic indicate that women may be more inclined than men to seek psychological support [62]. According to a study conducted in Türkiye, women had poorer mental health than men [23,39]. It has also been noted that women are more open to receiving psychological help [11,49]. In Türkiye, young women are less likely than young men to seek help. It is believed that the stigma associated with mental health disorders may have affected young women more. In Türkiye, seeking mental health treatment is viewed as an obstacle to marriage. The literature indicates that individuals with generalized anxiety disorder exhibit significantly higher levels of marital distress and a greater likelihood of divorce than those without the condition [63]. Due to concerns about stigmatization, it appears that the psychological help-seeking behaviour of young women may have been lower than that of young men. 

The survey year had an impact on psychological help-seeking. When analysed by year, the likelihood of people visiting a psychiatrist in 2019 is greater than in 2016, while young individuals were less likely to do so. While the rate of unhappiness in Türkiye in 2018 was 12.1%, it increased to 13.3% in 2019. Türkiye’s employment rate decreased in 2019 compared to the previous year and unemployment increased [64,65]. Increased unhappiness and unemployment may have contributed to a higher demand for psychological help in Türkiye. However, since most young people aged 15 to 24 are undergoing education at that age, this may have had a positive effect on this demographic. 

According to the study, young people between the ages of 15 and 24 are more likely to seek psychological help than those aged 25 and older. Studies indicate that younger age groups are more likely to seek psychological help [53]. Increasing age has been reported to exert a protective effect on psychological distress [55]. It has been demonstrated that as people age, their willingness to seek psychological help decreases [12]. It is reported that older individuals experiencing psychological distress often turn to informal sources for support [66]. These older individuals may seek help from family or friends rather than professional resources [54].

According to the study, individuals with health payment difficulties are more likely to seek psychological help. Individuals have difficulty paying for health services because they are unemployed. In this study, employment decreased psychological help-seeking. Unemployment is independently associated with more significant psychological distress [67]. Economic challenges and inadequate social support increase the probability of psychological distress [24,68,69]. Since unemployment may have caused financial difficulties, individuals’ mental health may have been negatively affected and they may have turned to seeking psychological help.

In this study, the education level affected the entire sample’s psychological help-seeking behaviour. In the entire sample, high school graduates were more likely to seek psychological help. Low education has been identified in the literature as a hindrance to seeking psychological help [53,54]. It has also been reported that depression literacy is significantly associated with attitudes toward seeking professional psychological help [12]. As mental health literacy increases, attitudes towards seeking psychological help improve positively [70]. In a systematic review of a group of migrants, it was found that individuals with a higher level of education had a more favourable attitude toward seeking psychological help [71]. Early treatment is crucial in safeguarding individuals from the consequences of mental health problems. Some individuals may prefer to address their issues independently rather than seek professional help [72]. Education plays a pivotal role in the battle against mental health issues. It can be argued that education significantly contributes to increasing awareness. However, in this study, the level of education in young people appears to have reduced their inclination to seek psychological help. School attendance and a steady job may have reduced the need for psychological support.

In this study, it was determined that married people were less likely to seek psychological help. Another study indicated that single individuals tend to be more open to seeking psychological help [53]. It has been reported that spousal support mitigates the negative psychological effects of unfair treatment [73]. This phenomenon may also be related to age. Social support from a spouse may be useful in reducing the number of married people visiting psychologists and psychotherapists.

In the study, those who perceived their general health to be in good shape sought less psychological help. Those with the disease and heart attack are more likely to seek psychological help. There exists a linear relationship between poor health status and psychological distress [74]. The person’s perception of their health has also been linked to psychological distress [75]. It has been reported that heart disease, migraine, bronchial asthma, and hypertension are associated with psychological distress [55]. Anxiety levels are higher in individuals with hypertension [76]. Some individuals with chronic diseases may reject the treatment burden [77,78]. The presence of a disease can increase stress and its impact on one’s well-being [79,80]. Moreover, the progression of a disease may also increase the stress caused by the disease, prompting more people to seek psychological help. Conditions such as the burden of disease and its treatment, changes in lifestyle, limitation, dependence on others, deterioration in the quality of life, and fatigue may intensify psychological distress and motivate individuals to seek psychological help [81,82,83]. Acute myocardial infarction is an unexpectedly fatal condition, and patients who experience a heart attack are susceptible to developing symptoms of post-traumatic stress disorder [84,85]. Consequently, individuals with post-traumatic psychological disorders may actively seek psychological help.

The behaviour of seeking psychological help is more prevalent throughout the entire sample in individuals who use tobacco and alcohol. Among young people, alcohol users are more likely to seek psychological help. Substance abuse is known to be detrimental to mental health [28] and is a globally recognized public health concern. A portion of substance abusers feel compelled to quit [86]. Individuals may have sought psychological help due to the substance’s negative impact on their mental health and the need to become free of it.

In the entire sample, individuals with few or no reliable relatives are more likely to seek psychological help. However, for young people, the situation is exactly the opposite. Interestingly, this study found that young people who received assistance from their neighbours were less likely to seek psychological help. Traumatic experiences hurt the mental health of individuals. Loneliness is directly related to psychological distress [56,57]. Such traumatic experiences often prompt people to seek emotional and social support [87]. In a study focusing on a group of suicidal individuals published in the scientific literature, survivors without social support may avoid contacting a psychologist [88]. When confronted with a dire circumstance, the absence of someone they can rely on impacts their mental health [23]. It has been reported that the measures taken during a pandemic can diminish the perception of social support, potentially leading to an increase in acute stress symptoms [89]. Both loneliness and traumatic events may motivate people to seek psychological help. It is possible that social support can alleviate psychological distress because it protects individuals from isolation.

The study data is from the pre-pandemic period. Individuals’ stressors increased during the pandemic compared to previous periods [90]. The spread of COVID-19 and related containment measures were significant stressors for most of the world’s population, with significant physical and mental health consequences [91]. It has been reported that prolonged exposure to significant challenges may lead to greater vulnerability in individuals [90].

It is essential to comprehend the factors that motivate people to seek psychological help in societies to determine the measures to be taken and the nature of the interventions to be implemented when global health problems such as pandemics arise.

## 6. Conclusions

Health is dynamic, and all government departments, not just the Ministry of Health, are responsible for preserving the community’s mental health. A multi-sectoral strategy is imperative to protect the community’s mental health, as individual, family, and community well-being are all influenced by a variety of circumstances. Human beings are biological, psychological, and sociological entities; therefore, various factors can affect mental health. While eliminating these factors may not be possible, people can be taught how to cope with those that negatively impact their mental health.

### 6.1. Practical Implications

As a result of this study, the preferences of individuals living in Türkiye regarding receiving support from psychologists, psychotherapists and psychiatrists are shown to be affected by gender, age, years of work, education, marital status, employment status, general health and disease status, previous heart attack, difficulty in paying for health services, and tobacco use. It has been found that alcohol use, having a reliable relative, and getting help from neighbours also have an effect. In light of these findings, it can be recommended that women, young people, those with economic difficulties, singles, and those living alone should be given priority in psychological support services. In future studies, it may be necessary to increase the number of experimental studies on women, the poor, and the lonely.

### 6.2. Theoretical Contributions

The findings of the multivariate analysis indicate that the various options for seeking psychological help are interrelated, and that these alternatives should be evaluated collectively. In this study, variables that may have an effect on the choice of seeing a psychologist, psychotherapist or psychiatrist were selected by considering the factors that may cause psychological distress in individuals. The results also show that the choice of psychological help-seeking alternatives is affected by many factors. These variables should be taken into consideration when creating new research topics.

### 6.3. Policy Implications

Wellness classes can be offered in primary care settings to improve overall health. These classes can provide training in nutrition, physical activity, quitting substance use, and stress management for individuals and groups. Public health nurses should educate patients on managing their illness and assessing their living environment to improve the quality of life of individuals with chronic diseases. The number of public health nurses with specialized training should also be increased in addition to the existing family health personnel in family medicine. Preventing substance use disorder and identifying those at risk for substance abuse is crucial; at risk individuals should be screened using substance-use disorder risk screening tools and be taught how to address their risk. Health professionals, such as physicians and nurses, who are trained in appropriate strategies should also be able to refer those with problematic substance use for treatment. A screening, brief intervention, and referral system may be recommended for this purpose.

To protect and promote community mental health, studying the systems of countries with high rates of happiness may prove beneficial. Resources must be expanded, and a community-based service model must be developed to preserve the community’s mental health. To effectively combat mental illness, inter-sectoral cooperation and cross-sector collaboration are necessary. Mandatory training to improve communication and counselling abilities may be recommended to strengthen the skills of personnel working in mental health services.

### 6.4. Limitations and Future Research

This study has several limitations. Firstly, the data in this study were secondary, and the variables necessary for statistical analysis were based on the available dataset. Secondly, as the data are cross-sectional, the causal relationship between psychological help and related socio-economic factors cannot be inferred. Thirdly, the data on questions about receiving psychological assistance services to individuals were the individuals’ answers. Therefore, the data obtained in this data collection method may be biased.

In future studies to protect mental health, experimental studies can be conducted on women, the poor, and lonely individuals. It is also recommended that new studies be planned to determine the coping mechanisms of this group to protect their mental health. Different methods and variables can be used to determine the variables that affect individuals’ preferences for psychologists or psychiatrists. Experimental studies must be planned to teach individuals and young people about receiving psychological help. Although visiting a psychologist, psychotherapist, or psychiatrist may occur due to psychological discomfort at different levels, it occurs due to psychological distress. Therefore, studies need to examine the relationship between psychological distress and preferences for seeing a psychologist, psychiatrist, or psychotherapist.

## Figures and Tables

**Table 1:** The Prevalence of individuals receiving psychological help services.

Dependent Variables	Frequency		Percentage	
	No	Yes	No	Yes
Psychologist	33,495	831	97.6	2.4
Psychotherapist	34,045	281	99.2	0.8
Psychiatrist	32,873	1453	95.8	4.2

**Table 2:** Findings related to factors affecting individuals’ receiving psychological help services.

Variables and Categories		Number	Percentage
Survey Year	2016	17,242	50.2
	2019	17,084	49.8
Gender	Male	15,452	45.0
	Female	18,874	55.0
Age	15–24	5635	16.4
	25 and above	28,691	83.6
Treatment Cost SSI	Yes	30,237	88.1
	No	4089	11.9
Education	Illiterate/No Degree	4842	14.1
	Primary School	5941	17.3
	Elementary School	6352	18.5
	High School	5631	16.4
	University	11,560	33.7
Marital Status	Single	10,688	31.1
	Married	23,638	68.9
Employment	Unemployed	21,342	62.2
	Employed	12,984	37.8
General Health Status	Very Good/Good	20,262	59.0
	Moderate	10,115	29.5
	Poor/Very Poor	3949	11.5
Disease Status	No	15,507	45.2
	Yes	18,819	54.8
Heart Attack	No	33,470	97.5
	Yes	856	2.5
Difficulty in Paying for Health	No	30,729	89.5
	Yes	3597	10.5
Tobacco Use	No	24,020	70.0
	Yes	10,306	30.0
Alcohol Consumption	No	29,725	86.6
	Yes	4601	13.4
Reliable Close Relative	None	1899	5.5
	1–2	12,897	37.6
	3 or more	19,530	56.9
Receiving Help from Neighbour	Very Easy/Easy	24,760	72.1
	Moderate	5272	15.4
	Difficult/Very Difficult	4294	12.5

**Table 3:** Predicted multivariate probit model for psychological help-seeking status.

Variables	Psychologist		Psychotherapist		Psychiatrist	
	β	Std. Error	β	Std. Error	β	Std. Error
Constant	−2.572	0.103	−3.213	0.159	−2.426	0.086
Gender (reference category: male)						
Female	0.314 ^a^	0.039	0.174 ^a^	0.057	0.272 ^a^	0.032
Survey Year (reference category: 2016)						
2019	−0.002	0.034	−0.023	0.051	0.091 ^a^	0.028
Youth-Adult (reference category: 25+)						
15–24	0.841 ^a^	0.316	0.836 ^c^	0.471	0.717 ^a^	0.270
Treatment Cost SSI (reference category: no)						
Yes	0.142 ^b^	0.060	0.169 ^c^	0.092	0.180 ^a^	0.050
Education (reference category: illiterate/no degree)						
Primary School	0.242 ^a^	0.051	0.288 ^a^	0.082	0.286 ^a^	0.043
Elementary School	0.235 ^a^	0.071	0.157	0.119	0.368 ^a^	0.056
High School	0.320 ^a^	0.065	0.508 ^a^	0.097	0.423 ^a^	0.053
University	0.417 ^a^	0.066	0.520 ^a^	0.101	0.467	0.055
Marital Status (reference category: single)						
Married	−0.100 ^b^	0.039	−0.140 ^b^	0.058	−0.133 ^a^	0.032
Employment Status (reference category: unemployed)						
Employed	−0.031	0.040	−0.013	0.060	−0.134 ^a^	0.033
General Health Status (reference category: poor/very poor)						
Very Good/Good	−0.352 ^a^	0.054	−0.273 ^a^	0.080	−0.406 ^a^	0.044
Moderate	−0.181 ^a^	0.046	−0.178 ^a^	0.069	−0.210 ^a^	0.038
Disease Status (reference category: no)						
Yes	0.347 ^a^	0.048	0.382 ^a^	0.073	0.457 ^a^	0.039
Heart Attack (reference category: no)						
Yes	0.165 ^b^	0.079	0.231 ^b^	0.106	0.001	0.070
Difficulty in Paying for Health (reference category: no)						
Yes	0.236 ^a^	0.045	0.320 ^a^	0.063	0.280 ^a^	0.038
Tobacco Use (reference category: no)						
Yes	0.157 ^a^	0.038	0.165 ^a^	0.055	0.212 ^a^	0.031
Alcohol Consumption (reference category: no)						
Yes	0.099 ^c^	0.052	−0.048	0.080	0.109 ^a^	0.042
Reliable Close Relative (reference category: 3 or more)						
None	0.243 ^a^	0.064	0.288 ^a^	0.093	0.071	0.058
1–2	0.090 ^b^	0.035	0.203 ^a^	0.053	0.059 ^b^	0.029
Receiving Help from Neighbour (reference category: difficult/very difficult)						
Very Easy/Easy	−0.077	0.048	0.045	0.075	−0.041	0.041
Moderate	−0.033	0.060	0.065	0.091	−0.045	0.050
Youth*Gender (reference category: male)						
Female	−0.258 ^b^	0.101	0.002	0.157	−0.195 ^b^	0.083
Youth*Survey Year (reference category: 2016)						
2019	−0.033	0.094	−0.057	0.143	−0.152 ^b^	0.077
Youth*Treatment Cost SSI (reference category: no)						
Yes	−0.157	0.131	0.221	0.262	−0.157	0.108
Youth*Education (reference category: illiterate/no degree)						
Primary School	−0.946 ^b^	0.452	−0.503	0.451	−0.317	0.269
Elementary School	−0.280	0.229	−0.607 ^c^	0.331	−0.337 ^c^	0.198
High School	−0.269	0.232	−0.655 ^b^	0.316	−0.235	0.201
University	−0.386	0.253	−0.677 ^c^	0.354	−0.227	0.216
Youth*Marital Status (reference category: single)						
Married	−0.089	0.149	−0.250	0.270	0.009	0.117
Youth*Employment Status (reference category: unemployed)						
Employed	−0.050	0.114	−0.124	0.181	−0.087	0.097
Youth*General Health Status (reference category: poor/very poor)						
Very Good/Good	−0.048	0.216	−0.113	0.322	−0.163	0.177
Moderate	0.002	0.221	−0.089	0.336	−0.191	0.183
Youth*Disease Status (reference category: no)						
Yes	0.155	0.109	0.010	0.168	0.087	0.089
Youth*Heart Attack (reference category: no)						
Yes	−0.044	0.576	−3.597	442.899	0.536	0.457
Youth*Difficulty in Paying for Health (reference category: no)						
Yes	0.114	0.131	−0.306	0.237	−0.067	0.115
Youth*Tobacco Use (reference category: no)						
Yes	0.052	0.110	0.060	0.172	0.000	0.090
Youth*Alcohol Consumption (reference category: no)						
Yes	0.140	0.127	0.092	0.212	0.271 ^a^	0.104
Youth*Reliable Close Relative (reference category: 3 or more)						
None	−0.221	0.189	−3.678	136.231	0.266 ^c^	0.150
1–2	−0.164 ^c^	0.100	−0.050	0.144	0.069	0.080
Youth*Receiving Help from Neighbour (reference category: difficult/very difficult)						
Very Easy/Easy	−0.240 ^b^	0.114	−0.254	0.191	−0.135	0.100
Moderate	−0.384 ^b^	0.159	−0.090	0.227	−0.018	0.124

^a^ *p* < 0.01; ^b^ *p* < 0.05; ^c^ *p* < 0.10.

**Table 4:** Marginal effects for psychological help seeking status.

Variables	Psychologist		Psychotherapist		Psychiatrist	
	ME(%)	Std. Error	ME(%)	Std. Error	ME(%)	Std. Error
Gender (reference category: male)						
Female	1.583 ^a^	0.002	0.337 ^a^	0.001	2.202 ^a^	0.003
Survey Year (reference category: 2016)						
2019	−0.010	0.002	−0.047	0.001	0.760 ^a^	0.002
Youth-Adult (reference category: 25+)						
15–24	8.341 ^c^	0.051	3.854	0.040	9.283 ^c^	0.050
Treatment Cost SSI (reference category: no)						
Yes	0.674 ^a^	0.003	0.291 ^b^	0.001	1.347 ^a^	0.003
Education (reference category: illiterate/no degree)						
Primary School	1.388 ^a^	0.003	0.664 ^a^	0.002	2.607 ^a^	0.004
Elementary School	1.459 ^a^	0.005	0.364	0.003	3.815 ^a^	0.007
High School	2.080 ^a^	0.005	1.548 ^a^	0.004	4.465 ^a^	0.007
University	2.922 ^a^	0.006	1.656 ^a^	0.005	5.114 ^a^	0.008
Marital Status (reference category: single)						
Married	−0.545 ^b^	0.002	−0.298 ^b^	0.001	−1.164 ^a^	0.003
Employment Status (reference category: unemployed)						
Employed	−0.162	0.002	−0.026	0.001	−1.082 ^a^	0.003
General Health Status (reference category: poor/very poor)						
Very Good/Good	−1.883 ^a^	0.003	−0.553 ^a^	0.002	−3.460 ^a^	0.004
Moderate	−0.924 ^a^	0.002	−0.345 ^a^	0.001	−1.706 ^a^	0.003
Disease Status (reference category: no)						
Yes	1.652 ^a^	0.002	0.670 ^a^	0.001	3.437 ^a^	0.003
Heart Attack (reference category: no)						
Yes	1.001 ^b^	0.005	0.589 ^c^	0.003	0.005	0.006
Difficulty in Paying for Health (reference category: no)						
Yes	1.457 ^a^	0.003	0.836 ^a^	0.002	2.770 ^a^	0.004
Tobacco Use (reference category: no)						
Yes	0.874 ^a^	0.002	0.355 ^a^	0.001	1.900 ^a^	0.003
Alcohol Consumption (reference category: no)						
Yes	0.559 ^c^	0.003	−0.092	0.001	0.971 ^b^	0.004
Reliable Close Relative (reference category: 3 or more)						
None	1.562 ^a^	0.005	0.773 ^b^	0.003	0.627	0.005
1–2	0.482 ^b^	0.002	0.427 ^a^	0.001	0.501 ^b^	0.002
Receiving Help from Neighbour (reference category: difficult/very difficult)						
Very Easy/Easy	−0.419	0.003	0.088	0.001	−0.347	0.003
Moderate	−0.170	0.003	0.136	0.002	−0.368	0.004
Youth*Gender (reference category: male)						
Female	−1.116 ^a^	0.004	0.004	0.003	−1.437 ^a^	0.005
Youth*Survey Year (reference category: 2016)						
2019	−0.169	0.005	−0.107	0.003	−1.145 ^b^	0.005
Youth*Treatment Cost SSI (reference category: no)						
Yes	−0.740	0.006	0.543	0.008	−1.194	0.007
Youth*Education (reference category: illiterate/no degree)						
Primary School	−2.189 ^a^	0.003	−0.589 ^b^	0.003	−2.062	0.013
Elementary School	−1.182	0.008	−0.709 ^a^	0.002	−2.243 ^b^	0.010
High School	−1.139	0.008	−0.774 ^a^	0.002	−1.668	0.012
University	−1.448 ^b^	0.006	−0.703 ^a^	0.002	−1.598	0.013
Youth*Marital Status (reference category: single)						
Married	−0.431	0.007	−0.380	0.003	0.076	0.010
Youth*Employment Status (reference category: unemployed)						
Employed	−0.250	0.005	−0.217	0.003	−0.679	0.007
Youth*General Health Status (reference category: poor/very poor)						
Very Good/Good	−0.243	0.011	−0.204	0.005	−1.237	0.012
Moderate	0.010	0.012	−0.162	0.006	−1.387	0.011
Youth*Disease Status (reference category: no)						
Yes	0.928	0.007	0.020	0.003	0.773	0.008
Youth*Heart Attack (reference category: no)						
Yes	−0.221	0.028	−0.772 ^a^	0.000	6.738	0.079
Youth*Difficulty in Paying for Health (reference category: no)						
Yes	0.667	0.008	−0.440 ^b^	0.002	−0.532	0.009
Youth*Tobacco Use (reference category: no)						
Yes	0.289	0.006	0.127	0.004	−0.003	0.008
Youth*Alcohol Consumption (reference category: no)						
Yes	0.834	0.009	0.204	0.005	2.783 ^b^	0.013
Youth*Reliable Close Relative (reference category: 3 or more)						
None	−0.952	0.007	−0.781 ^a^	0.000	2.733	0.018
1–2	−0.755 ^c^	0.004	−0.096	0.003	0.610	0.007
Youth*Receiving Help from Neighbour (reference category: difficult/very difficult)						
Very Easy/Easy	−1.059 ^b^	0.004	−0.405 ^c^	0.002	−1.034	0.007
Moderate	−1.445 ^a^	0.004	−0.163	0.004	−0.145	0.010

^a^ *p* < 0.01; ^b^ *p* < 0.05; ^c^ *p* < 0.10.

**Table 5:** Estimated correlation coefficients of dependent variables.

	Psychologist	Psychotherapist
Psychotherapist	0.634 *	
	(0.030)	
Psychiatrist	0.455 *	0.483 *
	(0.019)	(0.021)

* *p* < 0.05; Values in parentheses are standard errors.

## Data Availability

The Turkish Statistical Institute has restricted outside parties’ use of the study’s data. The Turkish Statistical Institute (bilgi@tuik.gov.tr) has data available for researchers who qualify for sensitive data. The study’s authors were not granted unique access rights to the data.
